# Adequate Vitamin D Intake but Low Serum Levels in Pediatric Asthma Patients: A Pilot Study, Alberta Children's Hospital

**DOI:** 10.1155/2016/6982010

**Published:** 2016-10-27

**Authors:** Sarah Howe McKenna, Tanis R. Fenton, Mary Noseworthy, Mark Anselmo

**Affiliations:** ^1^Alberta Children's Hospital, 2888 Shaganappi Trail NW, Calgary, AB, Canada T3B 6A8; ^2^Nutrition Services, Alberta Health Services, Calgary, AB, Canada; ^3^Alberta Children's Hospital Research Institute, O'Brien Institute of Public Health and Department of Community Health Sciences, Cumming School of Medicine, University of Calgary, Calgary, AB, Canada; ^4^Respirology, Alberta Children's Hospital, Cumming School of Medicine, University of Calgary, Calgary, AB, Canada

## Abstract

*Background*. We assessed vitamin D intakes and serum 25(OH) vitamin D levels in pediatric asthma patients on moderate-to-high dose inhaled steroids and compared them to published findings of healthy children in our city.* Methods*. Parents and/or patients were interviewed to estimate the children's vitamin D intakes from foods and supplements (using an adapted validated food frequency questionnaire) and asthma duration and management. Vitamin D status: serum 25-hyroxy vitamin D (25(OH)D) was obtained from the medical records.* Results*. Vitamin D intakes from food and supplements of the asthma patients (*n* = 20, 742 ± 185 IU/day) were significantly higher compared to healthy Canadian children (*n* = 1442, 229 ± 121 IU/day). Despite higher vitamin D intakes, the children had nonsignificantly lower serum 25(OH) vitamin D levels compared to the comparison group. Serum 25(OH)D levels increased by 3.6 nmol/L with each 100 IU of vitamin D intake (95% Confidence interval = 2.0–4.0, *R* ^2^ = 0.931, and *p* = 0.001).* Conclusion*. Since adequate vitamin D status in asthma patients is necessary to support bone mineral accretion, it is important to achieve adequate vitamin D status by checking serum 25(OH)D status and supplement accordingly.

## 1. Introduction

Asthma, a chronic inflammatory disease of the airway, is a common condition in Canada, with prevalence rates of approximately 8–10% in children [1]. While asthma can be managed medically, optimal management is not always achieved. Side effects of medications in children include growth restriction and bone disease [2–4].

Observational studies [5–7] and a randomized trial [8] have suggested that vitamin D could have a role in asthma management.

A systematic review of vitamin D randomized controlled trials in children with asthma found asthma exacerbation were less likely among the children randomized to vitamin D supplements of 500 to 2000 IU/day compared to placebo [9]. This finding is not based on strong evidence since three of the five included studies had unclear or no allocation concealment, so there is no assurance that the participants in the trials were appropriately randomized to the intervention arms. Data on other outcomes (including pulmonary function and asthma symptom scores) were heterogenous so meta-analysis was not possible. These findings need to be confirmed in larger well-designed randomized controlled trials [9].

There are several possible mechanisms of action for vitamin D in the asthmatic lung. For example, as pulmonary infection is a major asthma trigger, and vitamin D insufficiency has been associated with infections, it is possible that adequate vitamin D status is important for asthma control [10, 11].

As far as we are aware, no studies have examined vitamin D intakes in pediatric asthma patients or compared vitamin D intakes and the association between intakes and vitamin D status with that of normal children. The primary objective of the present study was to describe vitamin D intakes, from both food and supplements, in pediatric asthma clinic patients in Calgary and to compare these to the general population. A second objective was to determine whether children with severe asthma on high dose inhaled corticosteroids have lower vitamin D status (serum 25-hydroxy vitamin D (25(OH)D)) compared to healthy Canadian children [12]. A third objective was to determine if there was an association between vitamin D intake and serum 25(OH)D levels. The fourth objective was to determine whether vitamin D intake and status differed by asthma severity. The final objective was to determine if there was a relationship between vitamin D intake and serum 25(OH)D and asthma control.

## 2. Methods

### 2.1. Study Population and Design

The data was prospectively collected from severe asthma patients and/or their parents at an Alberta Children's Hospital Asthma Clinic appointment. The principal investigator attended asthma clinic once/week for 10 weeks to recruit patients. Inclusion criteria were age of 4–18 years and receiving an inhaled steroid dose of 400 micrograms per day or greater. All children who met the criteria were invited to participate and 100% agreed to do so.

The study was approved by the Conjoint Research Ethic Board at the University of Calgary. Written informed consent was provided by the patients and the parents or legal guardians of the patients. The principal investigator, a registered dietitian, interviewed the parent and/or patient between May and August, 2014, to ask about their consumption of vitamin D containing foods and use of vitamin supplements using a validated food frequency questionnaire [13], adapted for the Canadian food supply. Additionally, parents answered questions about asthma duration, asthma management, and use of a rescue inhaler. Information collected from the medical record included the child's age, sex, and vitamin D status: serum 25(OH)D. Ethical approval was granted for bloodwork done as part of routine care, only if the attending physician felt it was warranted. We recognize that this requirement could have induced a selection bias. Serum 25(OH)D levels were measured using Diasorin 25-OH Vitamin Total Assay. The serum 25(OH)D levels were compared to children in the same city from a study that prospectively collected them from healthy children who were having elective surgery over a one-year time period [12]. This study of healthy children used the same Diasorin 25-OH Vitamin Total Assay [12]. Asthma severity was defined as moderate if they were on 400/500 IU of inhaled corticosteroid and high if they were on 800 IU of corticosteroid. The measure of asthma control was reported frequency of rescue inhaler use. Vitamin D status was defined as sufficient when greater than 50 nmol/L, insufficient when between 37.5 and 50 nmol/L, and deficient when less than 37.5 nmol/L [14].

### 2.2. Statistical Analysis

Statistical comparisons between groups were made using *t*-tests for continuous variables and Fisher's exact test for categorical data, using an alpha level of 0.05 for statistical significance. Simple linear regression was used to examine the association between variables. We used post estimation dfbetas to determine whether the outlier was influential, using the rule that influence greater than one standard error indicated an influential result.

## 3. Results

Twenty children, aged 4–16 years, were invited and enrolled in the study and all completed the interview (Table 1). The participants were evenly divided by sex. Their mean age was 10.5 years (standard deviation (SD) = 3.4), and almost all (95%) had asthma for greater than 3 years. Their inhaled steroid medication regimens varied (Table 1).

A minority of the patients (*n* = 8, 40%) had vitamin D intakes (from food and supplements) within the recommended range, that is their intakes met or exceeded the Recommended Daily Allowance (RDA = 600 IU/day) and were below the Tolerable Upper Limit of 2000 IU/day (Table 2) [15]. Fifty-five percent (*n* = 11/20) of the patients did not meet the RDA for vitamin D and one patient's intake (5%) exceeded the Tolerable Upper Limit. The asthma patients obtained more vitamin D from their diets (including supplements) compared to healthy children assessed in the same city at the same time of year [12] (Table 2) (*p* < 0.0001). Analysis of the food frequency questionnaire revealed that 35% of the asthma patients (*n* = 7/20) and 90% of the healthy children (*n* = 1441/1586) or their parents reported the children consumed less than 400 IU vitamin D per day from food and supplements.

Serum 25-hydroxy vitamin D concentrations were available for only 7 (35%) study participants within 1 week of the interview. The majority of these patients (*n* = 5,71%) were vitamin D sufficient while two patients (29%) had serum 25(OH)D in the deficient range (<37.5 nmol/L). The one patient who exceeded the Tolerable Upper Limit for vitamin D intake had a serum vitamin D level (131 nmol/L) in the sufficient range [14]. Compared to healthy children in our city [12], these asthma patients had slightly, but not significantly, lower serum 25(OH)D levels (*p* = 0.07) (Table 2). Both the asthma patients in our study and the healthy children in our city [12] had 25(OH)D levels measured using the same method and at the same time of year.

**Figure 1:**
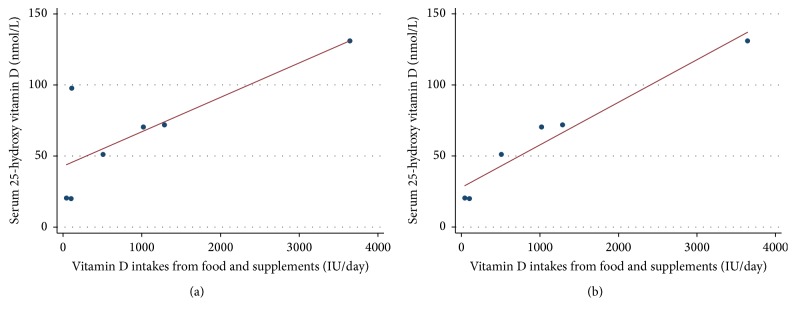
(a) Association between vitamin D intakes and status. Serum 25(OH)D levels increased by 2.4 nmol for every 100 IU of vitamin D intake (95% Confidence interval = 1.2–4.7, adjusted *R* ^2^ = 0.512, and *p* = 0.043). (b) Association between vitamin D intake and status without outlier. Without this outlier, serum 25(OH)D levels increased by 3.6 nmol for every 100 IU of vitamin D intake (95% Confidence interval = 2.0–4.0, adjusted *R* ^2^ = 0.931, and *p* = 0.001).

There was a significant association between vitamin D intakes from food and supplements with the participants' serum vitamin D levels (Figure 1). Serum 25(OH)D levels increased by 2.4 nmol for every 100 IU of daily vitamin D intake (95% Confidence interval = 1.2–4.7, adjusted *R* ^2^ = 0.512, and *p* = 0.043). In the graph illustrating this relationship, one child had a 25(OH)D level that was an apparent outlier with a considerably higher 25(OH)D than the other children (Figure 1(a)). This child had a noticeable suntan. The outlier decreased the beta-coefficient for the slope by 1.7 standard errors, so it was considered influential. Without this outlier, serum 25(OH)D levels increased by 3.6 nmol for every 100 IU of vitamin D intake (Figure 1(b), 95% Confidence interval = 2.0–4.0, adjusted *R* ^2^ = 0.931, and *p* = 0.001). Given this relationship, a patient with a serum level of 20 nmol/L could take a supplement of 1000 IU of vitamin D to raise the 25(OH)D level to 56 (20 + (3.6 × 1000/100) = 56) nmol/L over a 3-4 months' period [14].

The patients on 800 mcg of inhaled corticosteroids had lower serum vitamin D levels than those on 400/500 mcg; however, this difference was not significant (*p* = 0.62) (Table 2). The patients on 800 mcg also had lower vitamin D intakes (240 ± 189 IU/day) compared to those on 400/500 mcg (1499 ± 1322 IU/day) (*p* = 0.10).

Fifty-five percent of the patients used the rescue inhaler less than once/week, 20% used it 2–4 times per week, and 25% used it greater than twice per day. There was no relationship between adequate vitamin D intake and the use of the rescue inhaler, as their vitamin D intakes were evenly split between less than the DRI and more than the DRI (*p* = 1.00) for each category of rescue inhaler use. Similarly, there was no association between vitamin D sufficiency status and reduced use of the rescue inhaler with an even split between sufficient and insufficient intake and use of the rescue inhaler (*p* = 1.00).

## 4. Discussion

In this study of pediatric asthma patients receiving an inhaled steroid dose of 400 micrograms per day or greater, despite higher vitamin D intakes from food and supplements compared to healthy Canadian children in our city, we observed nonsignificantly lower serum 25(OH) vitamin D levels at the same time of year [12]. When compared to children in the Canadian Health Measures Survey [16], our patients had very similar mean 25(OH)D levels but this could be explained by seasonal variation. Our patients and our comparison population were all checked in the summer months; the Canadian Health Measures Survey measured vitamin D status in their participants in all seasons and did not differentiate between summer and winter serum 25(OH)D levels in their results.

Among these asthma patients, vitamin D intake and serum levels were closely related, with serum 25(OH)D levels, increasing by 3.6 nmol/L with each 100 IU of daily vitamin D intake, with 93% of the relationship explained by vitamin D intake. If found consistent in a larger study, this relationship could be used to determine vitamin D dosing for correction of low 25(OH)D levels in pediatric asthma patients.

A comprehensive systematic review on the rapid normalization of pediatric serum 25(OH)D vitamin D levels estimated from their multivariable analysis what doses would be required for rapid normalization of serum levels, considering children's age and baseline serum levels [17]. The systematic review authors estimated that vitamin D intakes close to the Tolerable Upper Intake Level (1000–4000 IU) brought children with deficient serum 25(OH)D to goal levels within a month [17].

While there have been studies conducted that have examined serum 25(OH) vitamin D levels in pediatric asthma patients [6, 7], as far as we are aware, this was the first study that examined both vitamin D intakes and serum 25(OH) vitamin D levels in this population.

Our study did not find a relationship between vitamin D intake or status with asthma control; however, our study was underpowered. A systematic review of vitamin D randomized controlled trials in children with asthma that observed a statistically significant benefit to asthma exacerbation (RR 0.41, CI 0.27–0.63, *p* < 0.0001) from vitamin D therapy was not able to conduct meta-analyses on pulmonary function tests due to variability of outcome measures used in the trials and missing data [9]. The systematic review's included studies were weak in terms of lack of objective criteria to define baseline disease severity and about how asthma was diagnosed or confirmed. One of the included studies found the placebo group had a greater reduction in asthma symptoms than did the vitamin D supplemented group. We agree with the systematic review authors that, prior to any conclusions being made, these findings need to be confirmed in larger well-designed randomized controlled trials.

Despite the current lack of consistent evidence for improved asthma control with adequate vitamin D, there may still be a need for vitamin D supplementation in pediatric asthma patients on moderate-to-high doses of corticosteroids, to support bone health. This group is at higher risk of decreased bone mineral density due to their corticosteroid use [18]. A study of asthmatic children followed over 4 years observed that oral corticosteroids were only negatively associated with poorer bone health among boys who were vitamin D insufficient, that is, serum 25(OH) vitamin D < 75 nmol/L. The vitamin D insufficient boys had two times lower bone mineral accretion compared to those who were vitamin D sufficient [19].

The patients in this study were all on greater than or equal to 400 mcg of inhaled corticosteroid, as this was one of the inclusion criteria. Higher inhaled corticosteroid use has been associated with increased risks of reduced bone mineral content bone fractures, as well as other systemic effects [20]. A relationship between high inhaled corticosteroid doses and low serum vitamin D status has been documented [6, 21]. Gupta et al. observed a relationship between high inhaled corticosteroid doses and low serum vitamin D status in children with moderate to severe treatment-resistant asthma [6]. They also observed that higher serum vitamin D levels were positively associated with improved lung function, better asthma control, and fewer asthma exacerbations in the previous six months. There was an association noted between daily corticosteroid use and lower vitamin D levels: the daily doses of inhaled corticosteroids steroid were inversely related to serum vitamin D levels. Our study did not find similar associations but our power was very low due the small sample size.

Inflammation is a major component of asthma. While it has been suggested that vitamin D may decrease inflammation in lung tissue [22], a randomized controlled trial by Bar Yoseph et al. showed no difference in airway inflammation between the vitamin D supplement group and the placebo group in children with mild asthma, aged 6–18 [23]. It is important to note that inflammatory processes may themselves lower serum 25(OH)D so low serum 25(OH) vitamin D may be a marker for inflammation [24]. This fact could explain both why patients with severe asthma have lower serum vitamin D levels compared to patients with mild or moderate asthma and why the participants in our study had lower 25(OH)D levels compared to the healthy Canadian children, despite having higher vitamin D intakes.

## 5. Conclusions

Our research supports the work of others [6, 7, 19], which together suggest there is good reason to check vitamin D levels in pediatric asthma patients on moderate-to-high doses of corticosteroids and to supplement them with this vitamin if their levels are suboptimal. Adequate vitamin D status in asthma patients is necessary to support bone mineral accretion; so it is important to achieve adequate vitamin D status. More research is needed to determine dosing for correction of suboptimal levels and maintenance as well as to determine the potential role that inflammation plays in suppressing vitamin D levels.

## Figures and Tables

**Table 1:** Patient variables.

Variables	*N* (%)
*Age*	
4–8 y	4 (20)
9–12 y	12 (60)
13–18 y	4 (20)
*Inhaled steroid dosage*	
Alvesco 400	9 (45)
Advair 400	1 (5)
Symbicort 400	2 (10)
Flovent 500	2 (10)
Symbicort 800	1 (5)
Alvesco 800	2 (10)
Zenhale 800	3 (15)
*Other medications*	
Singulair, 5 mg	10 (50)
Nasonex	1 (5)
Zolair	3 (15)
*Use of rescue inhaler*	
<1/week	11 (55)
2–4 × week	4 (20)
1/day	0
>2 × day	5 (25)

**Table 2:** Comparison of the study participants and healthy children in the same city and season.

	Asthma study participants in summer		Healthy local children in summer	
Age in years, *n* (%)	4–8	4 (20%)	2–5	788 (42)
	9–12	12 (60%)	5–9	727 (39)
	13–18	4 (20%)	9–13	347 (19%)

Ethnicity, *n* (%)	White	13 (65%)	White	1413 (77%)
	Asian	2 (10%)	Asian	163 (9%)
	African	3 (15%)	African	26 (1%)
	Others	2 (10%)	Others	271 (13%)

Serum 25(OH)D level (nmol/L) mean ± SD	66 ± 40^*∗*^ *n* = 7		89 ± 33^*∗*^ *n* = 1441	

Average vitamin D intake (IU/day) mean ± SD	742 ± 185^*∗∗*^ *n* = 20		229 ± 121^*∗∗*^ *n* = 1441	

*p* values: ^*∗*^ *p* = 0.07 and ^*∗∗*^ *p* < 0.0001.

## References

[1] http://www.asthma.ca/

[2] Chee C., Sellahewa L., Pappachan J. M. (2014). Inhaled corticosteroids and bone health. *Open Respiratory Medicine Journal*.

[3] Jung J.-W., Kang H.-R., Kim J.-Y., Lee S.-H., Kim S. S., Cho S. H. (2014). Are asthmatic patients prone to bone loss?. *Annals of Allergy, Asthma and Immunology*.

[4] Pruteanu A. I., Chauhan B. F., Zhang L., Prietsch S. O. M., Ducharme F. M. (2014). Inhaled corticosteroids in children with persistent asthma: dose-response effects on growth. *Evidence-Based Child Health*.

[5] Staple L. E., Teach S. J. (2011). Evidence for the role of inadequate Vitamin D in asthma severity among children. *Journal of Investigative Medicine*.

[6] Gupta A., Sjoukes A., Richards D. (2011). Relationship between serum vitamin D, disease severity, and airway remodeling in children with asthma. *American Journal of Respiratory and Critical Care Medicine*.

[7] Brehm J. M., Schuemann B., Fuhlbrigge A. L. (2010). Serum vitamin D levels and severe asthma exacerbations in the Childhood Asthma Management Program study. *Journal of Allergy and Clinical Immunology*.

[8] Majak P., Olszowiec-Chlebna M., Smejda K., Stelmach I. (2011). Vitamin D supplementation in children may prevent asthma exacerbation triggered by acute respiratory infection. *Journal of Allergy and Clinical Immunology*.

[9] Pojsupap S., Iliriani K., Sampaio T. Z. A. L. (2015). Efficacy of high-dose vitamin D in pediatric asthma: a systematic review and meta-analysis. *Journal of Asthma*.

[10] Charan J., Goyal J. P., Saxena D., Yadav P. (2012). Vitamin D for prevention of respiratory tract infections: a systematic review and meta-analysis. *Journal of Pharmacology and Pharmacotherapeutics*.

[11] Roth D., Shah R., Black R., Baqui A. (2010). Vitamin D status and acute lower respiratory infection in early childhood in Sylhet, Bangladesh. *Acta Paediatrica, International Journal of Paediatrics*.

[12] Stoian C. A., Lyon M., Cox R. G., Stephure D. K., Mah J. K. (2011). Vitamin D concentrations among healthy children in Calgary, Alberta. *Paediatrics and Child Health*.

[13] Nucci A. M., Russell C. S., Luo R. (2013). The effectiveness of a short food frequency questionnaire in determining vitamin D intake in children. *Dermato-Endocrinology*.

[14] Misra M., Pacaud D., Petryk A., Collett-Solberg P. F., Kappy M. (2008). Vitamin D deficiency in children and its management: review of current knowledge and recommendations. *Pediatrics*.

[15] National Research Council (2011). *Dietary Reference Intakes for Calcium and Vitamin D*.

[16] Canadian Health Measures Survey Data Tables http://www5.statcan.gc.ca/olc-cel/olc.action?ObjId=82-626-X&ObjType=2&lang=en&limit=0.

[17] McNally J. D., Iliriani K., Pojsupap S. (2015). Rapid normalization of vitamin D levels: a meta-analysis. *Pediatrics*.

[18] Kelly H. W., Van Natta M. L., Covar R. A., Tonascia J., Green R. P., Strunk R. C. (2008). Effect of long-term corticosteroid use on bone mineral density in children: a prospective longitudinal assessment in the childhood asthma management program (CAMP) study. *Pediatrics*.

[19] Tse S. M., Kelly H. W., Litonjua A. A., Van Natta M. L., Weiss S. T., Tantisira K. G. (2012). Corticosteroid use and bone mineral accretion in children with asthma: effect modification by vitamin D. *Journal of Allergy and Clinical Immunology*.

[20] Lipworth B. J. (1999). Systemic adverse effects of inhaled corticosteroid therapy: a systematic review and meta-analysis. *Archives of Internal Medicine*.

[21] Searing D. A., Zhang Y., Murphy J. R., Hauk P. J., Goleva E., Leung D. Y. M. (2010). Decreased serum vitamin D levels in children with asthma are associated with increased corticosteroid use. *Journal of Allergy and Clinical Immunology*.

[22] Banerjee A., Damera G., Bhandare R. (2008). Vitamin D and glucocorticoids differentially modulate chemokine expression in human airway smooth muscle cells. *British Journal of Pharmacology*.

[23] Bar Yoseph R., Livnat G., Schnapp Z. (2015). The effect of vitamin D on airway reactivity and inflammation in asthmatic children: a double-blind placebo-controlled trial. *Pediatric Pulmonology*.

[24] Autier P., Boniol M., Pizot C., Mullie P. (2014). Vitamin D status and ill health: a systematic review. *The Lancet Diabetes and Endocrinology*.

